# The G4 Genome

**DOI:** 10.1371/journal.pgen.1003468

**Published:** 2013-04-18

**Authors:** Nancy Maizels, Lucas T. Gray

**Affiliations:** 1 Department of Immunology, University of Washington, Seattle, Washington, United States of America; 2 Department of Biochemistry, University of Washington, Seattle, Washington, United States of America; Baylor College of Medicine, United States of America

## Abstract

Recent experiments provide fascinating examples of how G4 DNA and G4 RNA structures—aka quadruplexes—may contribute to normal biology and to genomic pathologies. Quadruplexes are transient and therefore difficult to identify directly in living cells, which initially caused skepticism regarding not only their biological relevance but even their existence. There is now compelling evidence for functions of some G4 motifs and the corresponding quadruplexes in essential processes, including initiation of DNA replication, telomere maintenance, regulated recombination in immune evasion and the immune response, control of gene expression, and genetic and epigenetic instability. Recognition and resolution of quadruplex structures is therefore an essential component of genome biology. We propose that G4 motifs and structures that participate in key processes compose the G4 genome, analogous to the transcriptome, proteome, or metabolome. This is a new view of the genome, which sees DNA as not only a simple alphabet but also a more complex geography. The challenge for the future is to systematically identify the G4 motifs that form quadruplexes in living cells and the features that confer on specific G4 motifs the ability to function as structural elements.

## G4 Motifs and G4 Structures

**Figure 1:**
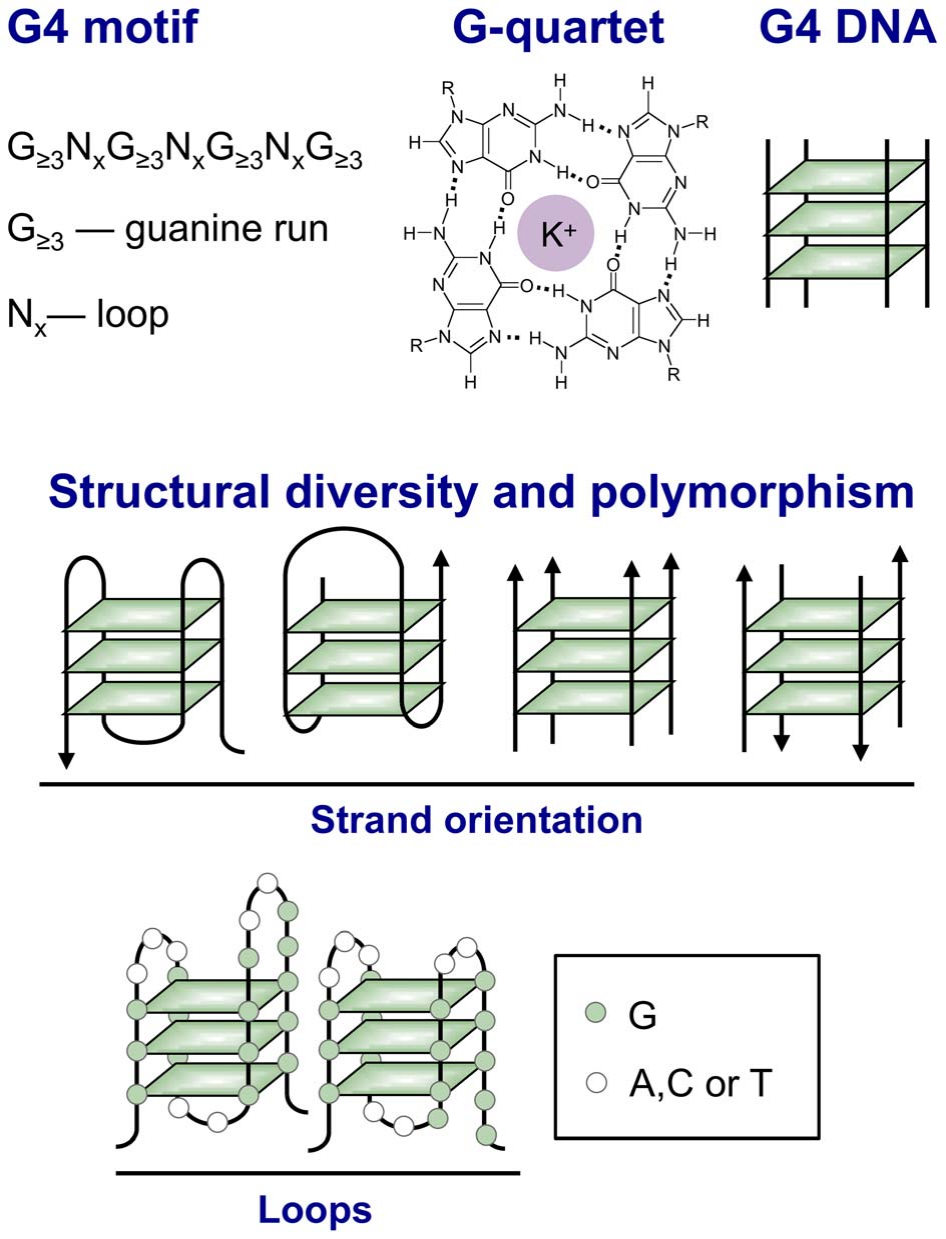
Structural variety of G4 DNA. Top: A G4 motif consists of four runs of at least three guanines per run, separated by other bases (N). G4 motifs confer the ability to form a G4 DNA structure, also known as a “quadruplex” by analogy with the *B*-form DNA duplex. The essential unit of G4 DNA is the G-quartet, a planar array of guanines stabilized by Hoogsteen base pairing between the N7 group of one guanine and the extracyclic amino group of its neighbor. The guanines form a ring around a central channel that is occupied by a monovalent cation and associated water molecules (potassium is shown). G4 structures derive stability both from hydrogen bonding between guanines within G-quartets and from stacking of the planar, hydrophobic G-quartets. Middle: The length and sequence composition of the loops connecting planar arrays of G-quartets (left) and the parallel (near right) or antiparallel (far right) orientation of nucleic acid strands determine quadruplex topology. Bottom: There is considerable potential for structural polymorphism, as illustrated by the diagram of two possible conformations formed by the G4 motif G_3_N_3_G_3_N_2_G_4_N_2_G_5_.

The sequence motif G_≥3_N*_x_*G_≥3_N*_x_*G_≥3_N*_x_*G_≥3_ confers the ability to form a four-stranded (“quadruplex”) structure in which interactions among strands are stabilized by G-quartets (Figure 1, see legend for details). G-quartets [1], G4 DNA [2], and G4 RNA [3] form readily in solution, creating structures of great variety [4], [5]. Strand orientation, conformation of glycosidic bonds of guanine bases in quartets, and sizes of and sequences of loops connecting the guanine runs contribute to structural intricacy. The diversity of G4 structures contrasts with the highly predictable *B*-form duplex. It has captivated chemists and fueled development of drugs that target quadruplexes and applications of quadruplexes to nanotechnology.

There are numerous G4 motifs in the human genome: over 350,000 allowing loops of 1–7 nt, and over 700,000 allowing loops up to 12 nt in length. G4 motifs are especially abundant in specific chromosomal domains, genomic regions, and genes. In human cells, the telomeres, rDNA, immunoglobulin switch regions (S regions), some variable number tandem repeats (VNTRs), and some single copy genes are all enriched for G4 motifs (“G4^hi^”). We do not yet know if every genomic G4 motif forms a quadruplex in a living cell. Nonetheless, G4 motifs provide a considerable potential repertoire for formation of diverse structures that may correlate with specific functions.

## G4 Motifs in DNA Replication

### DNA Replication Origins Coincide with G4 Motifs

Shared features of human DNA replication origins have been elusive. Origin identification by exhaustive high throughput sequencing of short nascent DNA strands has very recently revealed that the majority of the 250,000 human replication origins correspond to G4 motifs (67%, loop size 1–7 nt) [6]. This identifies half of the G4 motifs in the human genome as replication origins, and the fraction drops only slightly using more relaxed criteria for G4 motif identification. Origins proved to be highly conserved among four distinct human cell types: fibroblasts, embryonic stem cells, induced pluripotent stem cells, and HeLa cells. Quadruplexes are also implicated in recruitment of host factors for origin recognition by two human DNA viruses, SV40 [7] and Epstein-Barr virus [8]. It will be interesting to learn if components of the origin recognition complex recognize quadruplexes, and whether they discriminate among structures formed by distinct classes of G4 motifs.

### Quadruplexes and Telomere Maintenance in Human Cells

Human telomeres provide an example of both the utility and potential hazards of G4 motifs in DNA replication. They consist of thousands of kilobases of duplex DNA repeats (TTAGGG) and a single-stranded G-rich 3′-tail. Human telomeric repeats form characteristic “beads on a string” quadruplex structures, with a propeller-like domain [9], [10]. The importance of telomeres in cancer and aging has made them a paradigm for the design of G4-targeting drugs [11].

Human telomeres are transcribed from subtelomeric promoters to generate long, noncoding TERRA RNAs consisting of UUAGGG repeats, which adopt a characteristic G4 RNA structure [12], [13]. Use of a novel dual-pyrene probe specific for TERRA RNA G-quadruplexes has enabled direct imaging of TERRA G4 RNA at the telomeres in human cells [14]. The telomere binding protein TRF2 interacts with TERRA RNA G-quadruplexes to promote telomere heterochromatinization [15], [16]. Thus, G4 RNA is a key participant in telomere biology and epigenetic regulation.

G4 structures can protect telomeres. Telomeric repeats are normally capped by a protein complex that identifies them as telomeres rather than damaged DNA and protects them from misguided cellular efforts at repair that are potentially destabilizing [17]–[19]. In *Saccharomyces cerevisiae*, depletion of Cdc13, a component of the telomere-capping complex, results in telomere instability that can be countered by drugs that stabilize G4 structures [20]. G4 DNA and RNA are very resistant to digestion by exonucleases, and this may confer stability to telomeres deprived of caps. G4 structures may also facilitate t-loop formation, to establish the lariat conformation adopted by telomere ends in living cells.

### Replicative Instability at G4 Motifs

**Figure 2:**
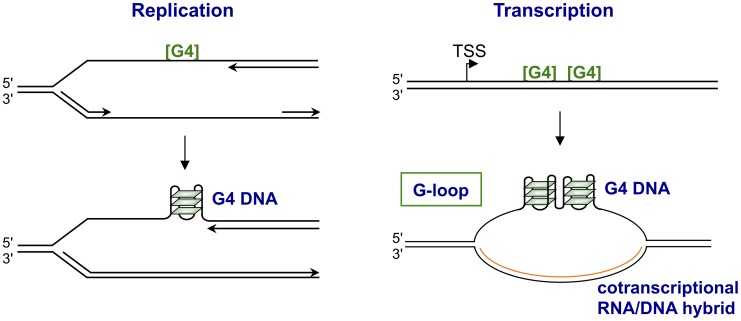
Structures form upon replication or transcription of regions bearing G4 motifs. The figures illustrate how replication (left) or transcription (right) through G4 motifs ([G4]) may result in formation of structures. Replication is shown as arresting at a G4 motif in the leading DNA strand, as it does in Pif1-deficient yeast [33]. Transcription is shown as resulting in formation of a G-loop, which contains a persistent RNA/DNA on the template strand and G4 DNA interspersed with single-stranded regions on the nontemplate strand [39].

Quadruplexes may form spontaneously when DNA single strands are exposed during replication or transcription (Figure 2). These structures pose challenges to replication, and G4 helicases must recognize and unwind G4 DNA to maintain genetic stability. G4 helicases in human cells include BLM [21], WRN [22], FANCJ [23], [24], CHL1 [25], PIF1 [26], and quite probably RTEL1 [18], [27]. The RecQ family G4 helicases WRN [28] and BLM [29] and the iron-sulfur domain helicase RTEL1 [30], [31] are required to resolve telomeric quadruplexes. Deficiency in WRN helicase occurs in the human genetic disease Werner syndrome, the most salient feature of which is premature aging due to depletion of telomeric sequence. Deficiency in BLM helicase causes Bloom syndrome, characterized by immunodeficiency due to impaired recombination at the G4^hi^ immunoglobulin S regions, as well as genomic instability and cancer predisposition. FANCJ deficiency is associated with instability at G4 motifs in human cells [23], [24], and causes an especially striking phenotype in *Caenorhabditis elegans*, evident as extended DNA deletions in which one end is bounded by a G4^hi^ region [32].

In *S. cerevisiae*, use of the G4^hi^ human CEB1 VNTR ( GGGGGGAGGGAGGGTGGCCTGCGGAGGTCCCTGGGCTGA) as a reporter identified Pif1 helicase as necessary for stability of G4 motifs [33], [34]. CEB1 instability was correlated with quadruplex structures by showing that a small molecule G4 ligand, PhenDC3, exacerbated instability, and that mutation that eliminated the potential to form quadruplexes conferred stability. Genomewide analysis of Pif1-deficient *S. cerevisiae* found endogenous G4 motifs enriched among sites of replication stalling [35]. Notably, instability associated with Pif1 deficiency exhibited an unexpected strand bias, and occurred if the G-rich strand was the template for leading (but not lagging) strand replication [34]. Helicases that unwind quadruplexes require an adjacent single-stranded region to load. The dependence of leading strand replication on Pif1, a 5′–3′ G4 helicase, may reflect the presence of exposed single-stranded region 5′ but not 3′ of a quadruplex structure on the leading strand, as new DNA synthesis may proceed to the very boundary of the quadruplex structure, thereby blocking the region where a 3′–5′ helicase would load [36].

## G4 Motifs in Regulated Recombination: Immune Evasion and the Immune Response

The most detailed examination of the biological function of a specific G4 DNA structure has been carried out in the course of defining the mechanism of pathogenesis of *Neisseria gonorrhoeae*. This obligate pathogen evades the human immune response by varying expression of its cell surface pilin proteins. Antigen variation depends upon gene conversion at the active pilin expression locus *pilE* using a reservoir of silent *pilS* loci as sequence donors. A specific recombination activator element functions *in cis* to regulate gene conversion. This element is a G4 motif (G_3_TG_3_TTG_3_TG_3_), and the element must form G4 DNA to promote variation [37]. Quadruplex formation requires transcription of the activator element from a dedicated upstream promoter, which generates a noncoding transcript [38]. RecA recognition of the *pilE* quadruplex stimulates strand exchange, but other G4 motifs do not support regulated recombination at the *pilE* locus [39]. Thus, protein–quadruplex recognition can be highly selective.

There are clear mechanistic analogies between immune evasion by *N. gonorrhoeae* and immunoglobulin gene class switch recombination in the vertebrate immune response. Class switch recombination is targeted to S regions, 2- to 8-kb repetitive G4^hi^ motifs, and deletes a long region of genomic DNA to join a new constant region to the expressed variable region, thereby altering the mode of antigen clearance without affecting antigen recognition. Each S region has a dedicated promoter, and transcription through the S region is necessary to activate recombination and target it to specific S regions. Transcription of S regions (and other G4^hi^ sequences) results in the formation of an unusual structure, a G-loop (Figure 2), containing a stable cotranscriptional RNA/DNA hybrid on the template strand and G4 DNA interspersed with single-stranded DNA on the nontemplate strand [40]–[42]. Quadruplexes formed in the transcribed S regions are the targets of factors that promote switch recombination, including BLM helicase, and MutSα (reviewed by [43]). MutSα also functions in telomere maintenance and in repair of DNA base mismatches and small loops. Human MutSα binds to quadruplexes in G-loops formed by transcribed S regions and can promote synapsis between distinct S regions in solution [44]. *Escherichia coli* MutS also binds quadruplexes [45], so quadruplex binding is a conserved property of this factor.

## G4 Motifs in Genes and Transcripts

### Characteristic Distribution of G4 Motifs within Genes

**Figure 3:**
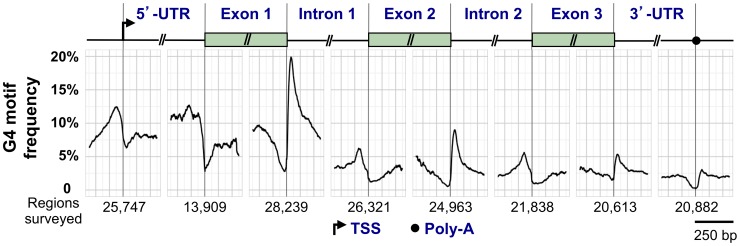
G4 motif frequency in a generic human RefSeq gene. Above, key elements of a generic gene are shown, including the TSS, 5′-UTR, 5′ exons and introns, and the 3′-UTR and poly(A) signal. Below, the graph shows the frequency of G4 motifs in each region (loop size 1–12 nt). G4 motif frequency was calculated by counting the number of times G4 motifs overlapped each position, and dividing by the number of regions surveyed, which varied for each window. G4 locations at the 5′-UTR–exon 1 boundary were calculated only for genes with a 5′-UTR that did not span multiple exons.

G4 motifs exhibit a characteristic distribution within human RefSeq genes (genomic sequences used as reference standards for well-characterized genes; http://www.ncbi.nlm.nih.gov/refseq/rsg/about/). Figure 3 diagrams a generic RefSeq gene, with the genomewide average of G4 motifs plotted relative to standard reference points including the transcription start site (TSS), 5′-UTR, exons, introns, and 3′-UTR. G4 motifs are enriched at the TSS, the 5′-UTR, and the 5′ end of the first intron, and depleted in coding regions. The coding regions of most genes are depleted for G4 motifs (G4^lo^), but some are enriched (G4^hi^) [46].

The regions flanking the TSS are G4^hi^ [47]–[50]. The presence of G4 motifs in promoters of oncogenes, such as MYC and RAS, fueled efforts to develop small molecule ligands that would bind to a postulated quadruplex and downregulate gene expression [51]. However, it has proved challenging to develop a ligand specific for a single quadruplex in the genome of a human cell, and this drug development strategy has not yet been fruitful.

Current evidence for regulation by binding of factors to quadruplexes near the TSS is relatively limited. Analysis of genomewide associations by chromatin immunoprecipitation sequencing (ChIP-Seq) can establish protein enrichment at specific sequence motifs, and this has provided one line of evidence that promoter quadruplexes may regulate transcription. In those experiments, the ubiquitous transcription factor SP1, which binds a G-rich consensus motif GGGCGG in duplex DNA, was shown to bind G4 DNA in solution, and to preferentially associate with G4 motifs at promoter regions in living cells [52].

G4 motifs are enriched downstream of the TSS in the nontemplate DNA strand, where they may form quadruplexes in either the DNA or RNA [48]. Functions of these quadruplexes are potentially interesting, but need better definition. In 10%–15% of human genes, a G4 motif occurs in the region specifying the 5′-UTR of the encoded mRNA, leading to the suggestion that G4 RNA structures may promote translational repression (reviewed by [53]). At many human genes, RNA Pol2 initiates transcription but pauses near the 5′ end, and pausing correlates with enrichment of G4 motifs [50]. In nearly half of all human genes, one or more G4 motifs are present at the very 5′ end of the first intron, on the nontemplate strand, and will be transcribed into the encoded pre-mRNA [54]. The G4^hi^ element in the first intron of the gene encoding the G4 helicase CHL1, has been shown to form a quadruplex structure not previously documented, and resembling the catalytic core of group I introns [55]. This RNA quadruplex may be the target of regulation, even by CHL1 itself, but this needs to be carefully studied.

Both quadruplexes and quadruplex binding proteins may function in 3′-end processing of mRNA transcripts. Cell stress regulates 3′-end processing of the P53 gene transcript, and altered 3′-end processing has been shown to depend upon recognition of a G4 RNA structure by hnRNP H/F [56]. More generally, RNA Pol2 must pause at a G4^hi^ region just downstream of the poly(A) site to enable transcriptional termination at a subset of genes in normally proliferating cells, to enable 3′-end processing by XRN nuclease and the DNA/RNA helicase senataxin [57]. Deficiencies in senataxin are associated with two neurological diseases, ataxia oculomoter apraxia 2 and amyotrophic lateral sclerosis type 4.

### Transcription-Induced Genomic Instability at R-Loops and G-Loops

Transcribed G4 motifs pose a special threat to genomic stability. Transcription of G-rich regions results in formation of R-loops, which are targets of transcription-associated genomic instability, as has been recently reviewed [58], [59]. Like G-loops (Figure 2), R-loops contain an RNA/DNA hybrid, but they do not necessarily contain the nontemplate strand G4 DNA that makes G-loops a target for some recombination factors [40]–[42]. Nonetheless, many of the regions that form cotranscriptional R-loops are G4^hi^, and the nontemplate strand is likely to contain quadruplexes that may contribute to biological function even if those structures are not explicitly acknowledged. For example, it has been reported that promoter regions that form R-loops are protected from de novo methylation at CpG dinucleotides [60]. The authors recognized that R-loop formation is a property of G-rich regions, but did not extend their analysis to include G4 motifs.

The contribution of transcription-induced G-quadruplexes with genomic instability has been established by genetic analyses, and by use of a G4 DNA ligand, pyridostatin [61]. Pyridostatin treatment of human cells resulted in transcription-dependent appearance of DNA damage markers, including γ-H2AX, and arrest in the G2 phase of the cell cycle. Genomewide analysis by ChIP-Seq showed that G4 motifs were enriched among sites of damage. Pyridostatin interacts with an exposed planar G-quartet rather than loop sequences in solution, suggesting that it might recognize a very broad spectrum of G4 structures in a living cell. However, damage induced by pyridostatin was restricted to a subset of G4^hi^ genes—including the actively transcribed rDNA in the nucleolus, but not the telomeres—and the SRC (but not HRAS) oncogene [61]. This selectivity, which was not anticipated by biochemical characterization of pyridostatin, points to the importance of validating predicted cellular targets of reagents directed at quadruplexes.

## Neurological Disease Associated with G4 Motif Repeat Expansions in Specific Genes

Expansions of G4 motifs in five different genes are associated with neurological disease (Table 1). Expansions in the FMR1 [62], C9orf72 [63]–[65], and NOP56 [66] genes produce pre-mRNAs that carry extended regions of quadruplex structures, which appear to titrate essential RNA binding proteins and impair mRNA processing. At the CTSB gene [67], the expanded CGCGGGGCGGGG repeat in the promoter is thought to promote excessive DNA methylation at CpG dinucleotides that downregulate gene expression.

**Table 1:** G4 motif expansions in neurological disease.

Repeat	Gene	Disease
CGG	FMR1, 5′-UTR	Fragile X syndrome, fragile X–associated tremor/ataxia syndrome
GGGGCC	C9orf72, intron 1	Amyotrophic lateral sclerosis (ALS4), autosomal dominant frontotemporal dementia
GGCCT	NOP56, intron 1	Spinocerebellar ataxia (SCA36)
CGCGGGGCGGG	CSTB, promoter	Progressive myoclonus epilepsy type 1
CCCCATGGTGGTGGCTGGGGACAG	PRNP, coding exon	Creutzfeldt-Jakob disease

Especially intriguing is an expansion in the coding region of the PRNP gene (Table 1), which encodes the prion protein associated with Creutzfeldt-Jakob disease [68]. The normal prion protein contains five repeats of the sequence CCCCATGGTGGTGGCTGGGGACAG. Expansions, typically to 10–14 repeats, cause a dominant form of familial Creutzfeldt-Jakob disease exhibiting early onset and slow progression, which correlate with misfolding of the corresponding prion protein and formation of insoluble protein aggregates in solution [69].

The mechanisms that drive expansions of these G4 motifs have not been defined. Transcription may promote instability, as suggested by the inherent instability of regions prone to form persistent cotranscriptional RNA/DNA hybrids. Expansion may reflect mitotic instability, dramatically evident at G4^hi^ VNTRs, which rank among the most unstable repeats in the human genome.

## Epigenetic Instability at G4 Motifs

Unimpeded replication of G4 motifs is important not only in genetic stability but also in epigenetic stability. Deficiency in FANCJ, BLM, or WRN helicases can cause alterations of epigenetic modifications near G4 motifs, evident as increased expression of silenced genes or reduced expression of active genes [70], [71]. This may reflect replication slowdown at G4 motifs that prevents the local redeposition of marked histones necessary to maintain epigenetic status [72].

If G4 motifs do contribute to epigenetic regulation by enabling epigenetic marks to be reset upon replication, then genes that respond rapidly to external stimuli would be predicted to be G4^hi^. This is in fact the case. Genomewide analyses have shown that the G4^hi^ or G4^lo^ status of a gene extends throughout its length, including both exons and introns, and that it correlates with gene function [46]. G4^hi^ genes include transcriptional activators, developmental regulators, and oncogenes, such as MYC, JUNB, FGF4, and TERT, which respond rapidly to developmental and environmental stimuli.

The possibility that G4 motifs may function as epigenetic regulatory elements is supported by analyses of ATRX, a SWI/SNF family member with robust G4 DNA binding activity [73]–[75]. ATRX is enriched at the G4^hi^ telomeres and rDNA, and at G4 motifs elsewhere throughout the genome, including a polymorphic G4^hi^ VNTR at the α-globin gene, CGCGGGGCGGGGG . Deficiencies in ATRX are associated with an X-linked genetic disease characterized by α-thalassemia and mental retardation. The severity of α-thalassemia resulting from ATRX deficiency correlates with α-globin VNTR length [75], recapitulating features of diseases associated with trinucleotide repeat instability [76].

Genomewide studies suggest that G4 motifs tend to be hypomethylated and depleted for nucleosomes, in normal cells and especially in human tumors, where hypomethylated G4 motifs predominate among sites of genomic instability leading to copy number variation [77]–[80]. The relatively relaxed state of hypomethylated chromatin may be conducive to quadruplex formation. Thus, epigenetic and genetic instability at G4 motifs may go hand in hand.

## Future Challenges: Defining the G4 Genome

### Which G4 Motifs Form Quadruplexes in Living Cells?

This question can be addressed by systematically defining genomewide targets of endogenous quadruplex binding proteins by ChIP-Seq, or by identifying targets of reagents with validated specificity for quadruplexes in living cells. Both approaches have been used in contexts noted above. For example, associations of SP1 in the human genome [52] and of Pif1 in the *S. cerevisiae* genome [35] have been mapped by ChIP-Seq, and the G4 ligands PhenDC3 and pyridostatin have been used to specifically exacerbate instability at quadruplexes in *S. cerevisiae* [33] and human cells [61]. Quite recently, a single chain antibody selective for quadruplexes in solution was shown to stain chromosomes in the nuclei of human cells [81]. If antibody specificity for quadruplexes in living cells can be validated (e.g., by ChIP-Seq), it will be a useful tool for studying the cell biology of quadruplexes.

### How Are Specific Quadruplexes Recognized to Enlist Participation in Specific Pathways?

The diverse conformations of quadruplexes suggest that specific structural features may enable participation in specific pathways. To take one example, about half the G4 motifs in the human genome map to replication origins. What distinguishes those quadruplexes? Co-crystal or nuclear magnetic resonance structures of protein/quadruplex complexes are essential to identify the structural determinants that attract specific proteins to specific quadruplexes.
